# Supplementary material for: Mitochondrial Genomes of Two Thaparocleidus Species (Platyhelminthes: Monogenea) Reveal the First rRNA Gene Rearrangement among the Neodermata
Source: Int J Mol Sci. 2019 Aug 28;20(17):4214. doi: 10.3390/ijms20174214 (PMC6747449; doi:10.3390/ijms20174214)
Supplement: Supplementary file 1 [file ijms-20-04214-s001.zip › ijms-573981-SI legends.docx]

**Supplementary Figure S1.** Relative synonymous codon usage (RSCU) of the mitogenomes of Thaparocleidus asoti and Thaparocleidus varicus. Codon families are labelled on the x-axis. Values on the top of the bars denote the amino acid usage.

**Supplementary Figure S2.** All four phylograms generated by mitogenomes and 28S rDNA sequences.

**Supplementary Table S1.** Summary of overlapping regions among the monogeneans.

**Supplementary Table S2.** Monogenean species used for the mitochondrial phylogenomics analyses.

**Supplementary Table S3.** General statistics for the protein-coding and rRNA genes of the 33 selected monogenean species.

**Supplementary Table S4.** Primers used to amplify and sequence the mitochondrial genomes of Thaparocleidus asoti and Thaparocleidus varicus.

**Supplementary Table S5.** Monogenean species and outgroups used for phylogenetic reconstruction based on the 28S rDNA sequences.
