# Supplementary material for: Mitochondrial Genomes of Two Thaparocleidus Species (Platyhelminthes: Monogenea) Reveal the First rRNA Gene Rearrangement among the Neodermata
Source: Int J Mol Sci. 2019 Aug 28;20(17):4214. doi: 10.3390/ijms20174214 (PMC6747449; doi:10.3390/ijms20174214)
Supplement: Supplementary file 1 [file ijms-20-04214-s001.zip › Supplementary Figure S1.pdf]

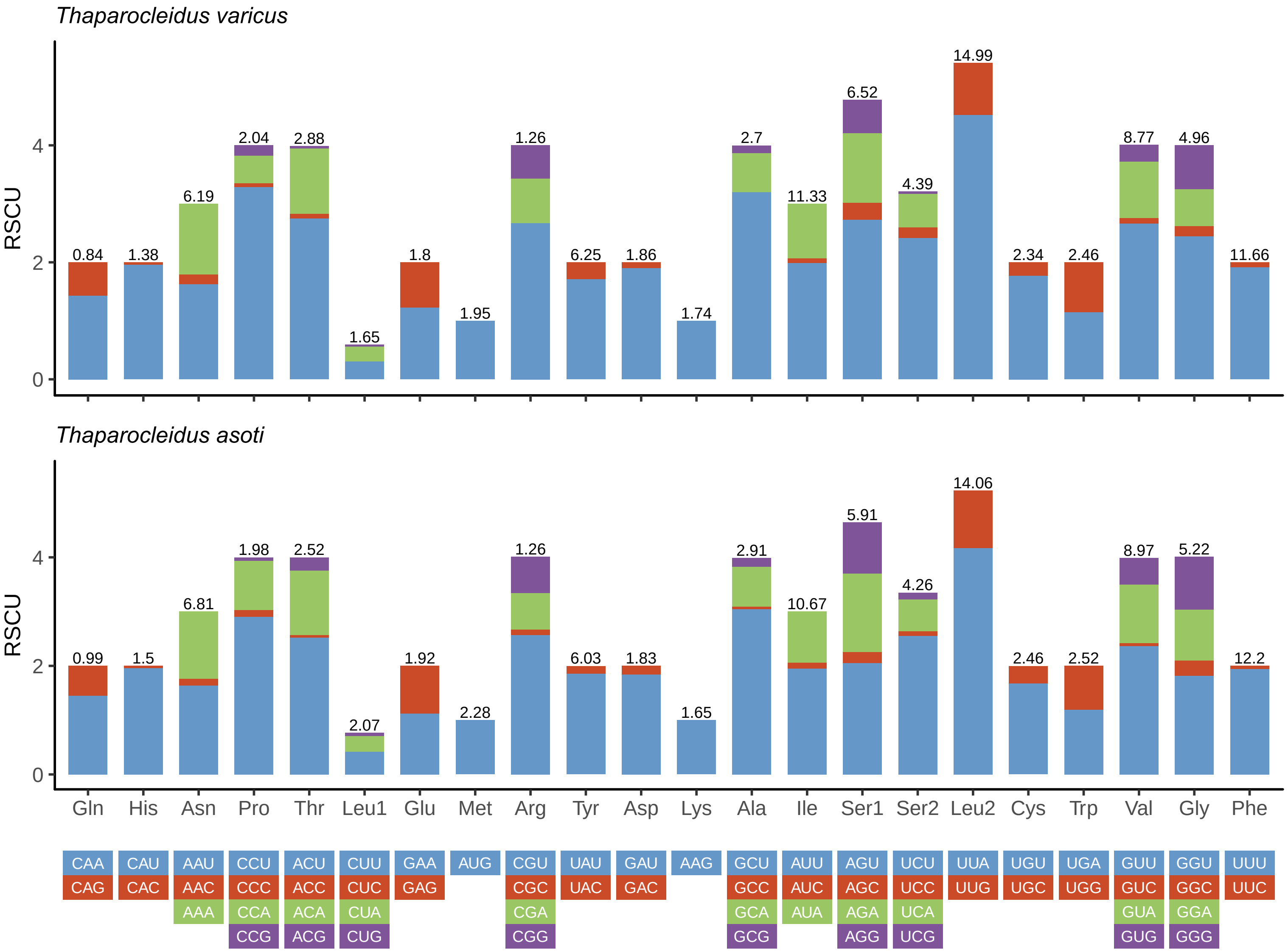
Thaparocleidus varicus
RSCU
0
2
4
0.84
1.38
6.19
2.04
2.88
1.65
1.8
1.95
1.26
6.25
1.86
1.74
2.7
11.33
6.52
4.39
14.99
2.34
2.46
8.77
4.96
11.66
Thaparocleidus asoti
0.99
1.5
6.81
1.98
2.52
2.07
1.92
2.28
1.26
6.03
1.83
1.65
2.91
10.67
5.91
4.26
14.06
2.46
2.52
8.97
5.22
12.2
Gln His Asn Pro Thr Leu1 Glu Met Arg Tyr Asp Lys Ala Ile Ser1 Ser2 Leu2 Cys Trp Val Gly Phe
CAA CAU AAU CCU ACU CUU GAA AUG CGU UAU GAU AAG GCU AUU AGU UCU UUA UGU UGA GUU GGU UUU
CAG CAC AAC CCC ACC CUC GAG CGC UAC GAC GCC AUC AGC UCC UUG UGC UGG GUC GGC UUC
AAA CCA ACA CUA CGA GCA AUA AGA UCA GUA GGA
CCG ACG CUG CGG GCG AGG UCG GUG GGG
