# Supplementary material for: Mitochondrial Genomes of Two Thaparocleidus Species (Platyhelminthes: Monogenea) Reveal the First rRNA Gene Rearrangement among the Neodermata
Source: Int J Mol Sci. 2019 Aug 28;20(17):4214. doi: 10.3390/ijms20174214 (PMC6747449; doi:10.3390/ijms20174214)
Supplement: Supplementary file 1 [file ijms-20-04214-s001.zip › Supplementary Figure S2.pdf]

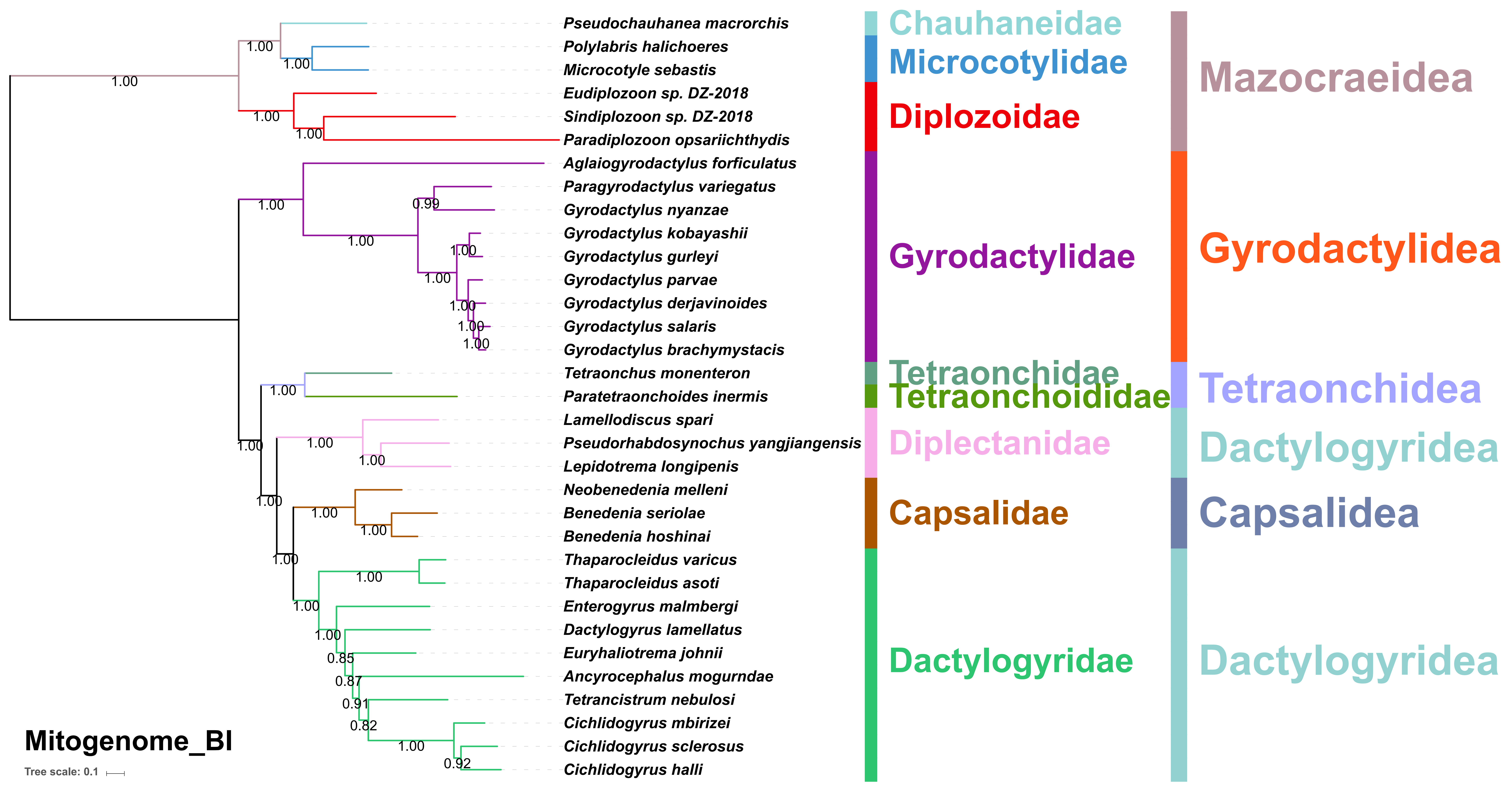

Pseudochauhanea macrorchis
Polylabris halichoeres
Microcotyle sebastis
Eudiplozoon sp. DZ-2018
Sindiplozoon sp. DZ-2018
Paradiplozoon opsariichthydis
Aglaiogyrodactylus forficulatus
Paragyrodactylus variegatus
Gyrodactylus nyanzae
Gyrodactylus kobayashii
Gyrodactylus gurleyi
Gyrodactylus parvae
Gyrodactylus derjavinoides
Gyrodactylus salaris
Gyrodactylus brachymystacis
Tetraonchus monenteron
Paratetraonchoides inermis
Lamellodiscus spari
Pseudorhabdosynochus yangjiangensis
Lepidotrema longipenis
Neobenedenia melleni
Benedenia seriolae
Benedenia hoshinai
Thaparocleidus varicus
Thaparocleidus asoti
Enterogyrus malmbergi
Dactylogyrus lamellatus
Euryhaliotrema johnii
Ancyrocephalus mogurndae
Tetrancistrum nebulosi
Cichlidogyrus mbirizei
Cichlidogyrus sclerosus
Cichlidogyrus halli
Chauhaneidae
Microcotylidae
Diplozoidae
Gyrodactylidae
Tetraonchidae
Tetraonchoididae
Diplectanidae
Capsalidae
Dactylogyridae
Mazocraeidea
Gyrodactylidea
Tetraonchidea
Dactylogyridea
Capsalidea
Dactylogyridea
1.00
0.99
0.85
0.87
0.91
0.82
0.92
Mitogenome_BI
Tree scale: 0.1

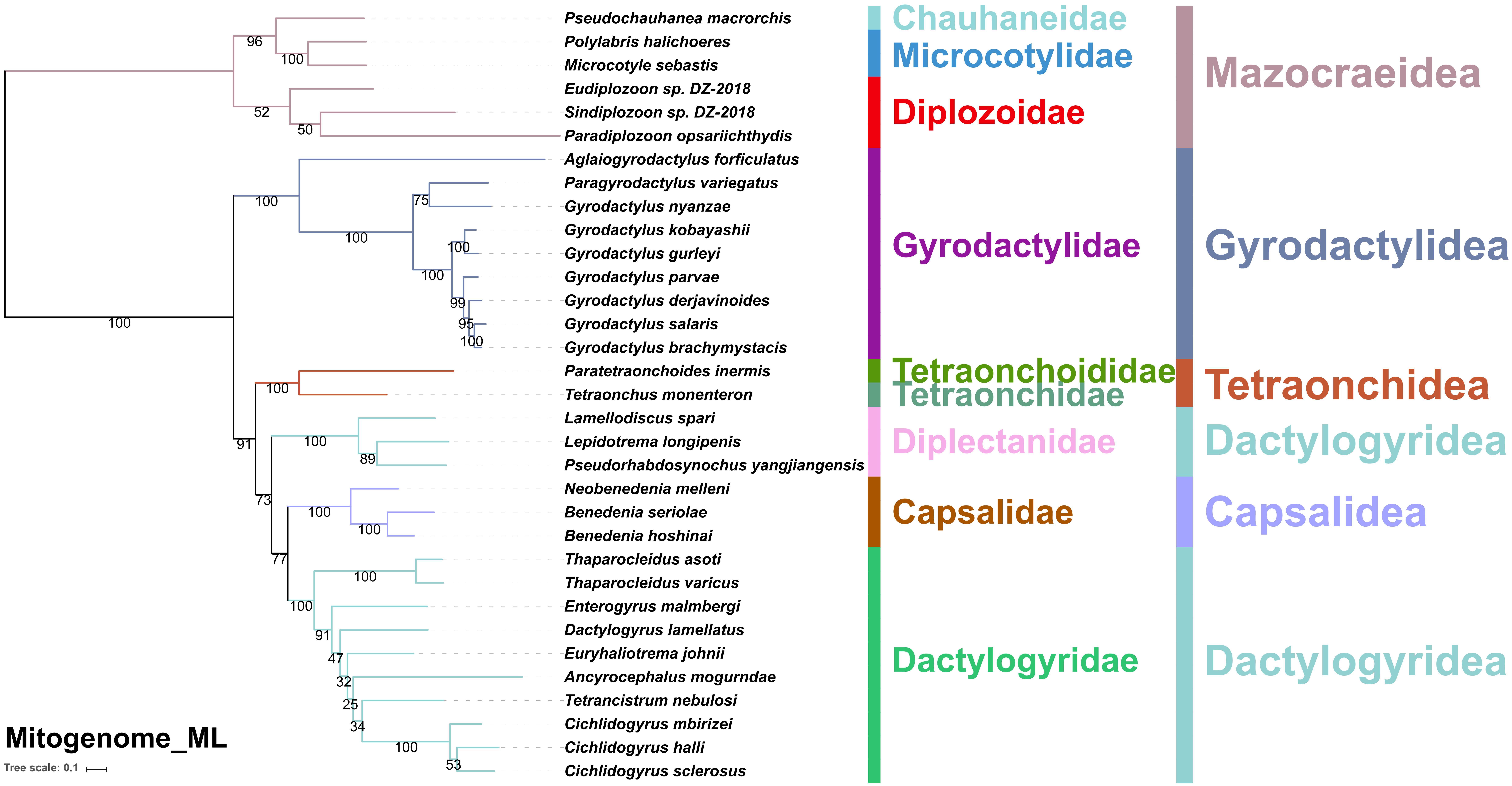

Pseudochauhanea macrorchis
Polylabris halichoeres
Microcotyle sebastis
Eudiplozoon sp. DZ-2018
Sindiplozoon sp. DZ-2018
Paradiplozoon opsariichthydis
Aglaiogyrodactylus forficulatus
Paragyrodactylus variegatus
Gyrodactylus nyanzae
Gyrodactylus kobayashii
Gyrodactylus gurleyi
Gyrodactylus parvae
Gyrodactylus derjavinoides
Gyrodactylus salaris
Gyrodactylus brachymystacis
Paratetraonchoides inermis
Tetraonchus monenteron
Lamellodiscus spari
Lepidotrema longipenis
Pseudorhabdosynochus yangjiangensis
Neobenedenia melleni
Benedenia seriolae
Benedenia hoshinai
Thaparocleidus asoti
Thaparocleidus varicus
Enterogyrus malmbergi
Dactylogyrus lamellatus
Euryhaliotrema johnii
Ancyrocephalus mogurndae
Tetrancistrum nebulosi
Cichlidogyrus mbirizei
Cichlidogyrus halli
Cichlidogyrus sclerosus
Chauhaneidae
Microcotylidae
Diplozoidae
Gyrodactylidae
Tetraonchoididae
Tetraonchidae
Diplectanidae
Capsalidae
Dactylogyridae
Mazocraeidea
Gyrodactylidea
Tetraonchidea
Dactylogyridea
Capsalidea
Dactylogyridea
Mitogenome_ML
Tree scale: 0.1

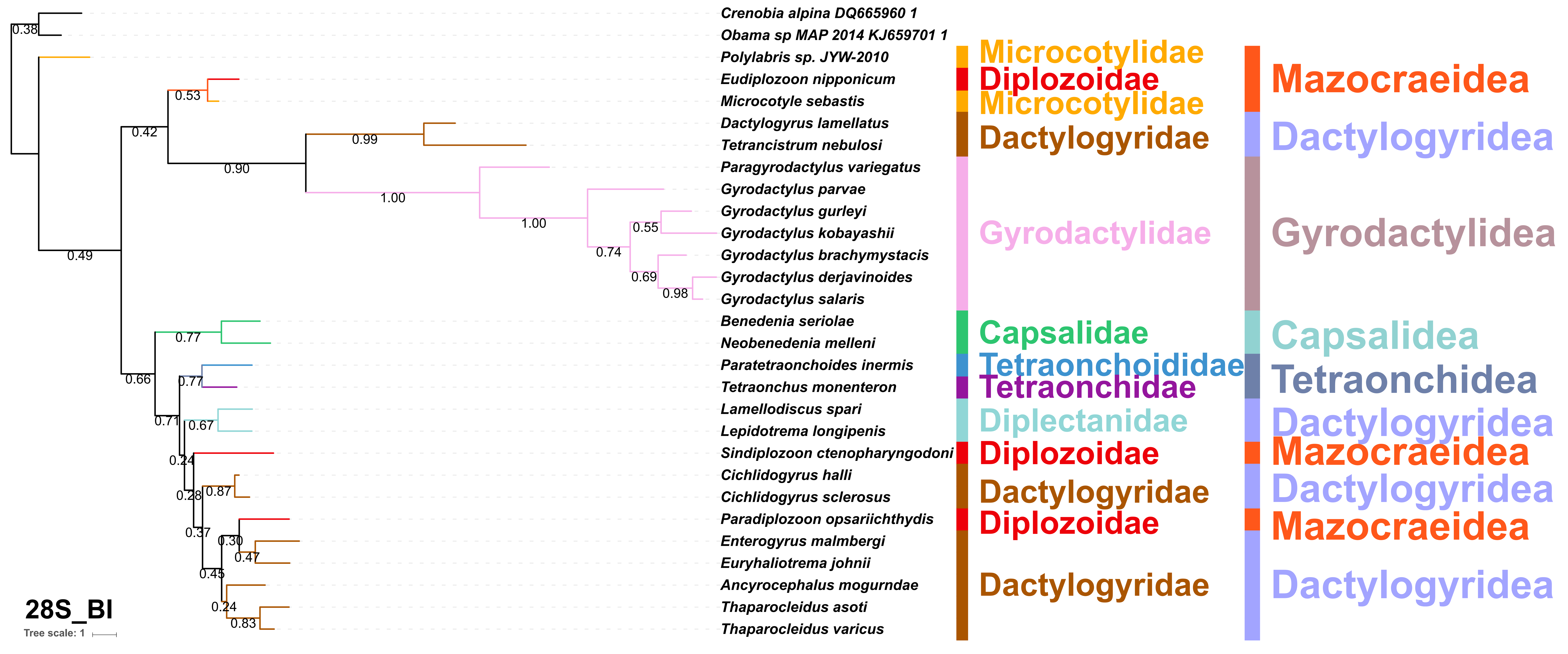
Crenobia alpina DQ665960 1
Obama sp MAP 2014 KJ659701 1
Polylabris sp. JYW-2010
Eudiplozoon nipponicum
Microcotyle sebastis
Dactylogyrus lamellatus
Tetrancistrum nebulosi
Paragyrodactylus variegatus
Gyrodactylus parvae
Gyrodactylus gurleyi
Gyrodactylus kobayashii
Gyrodactylus brachymystacis
Gyrodactylus derjavinoides
Gyrodactylus salaris
Benedenia seriolae
Neobenedenia melleni
Paratetraonchoides inermis
Tetraonchus monenteron
Lamellodiscus spari
Lepidotrema longipenis
Sindiplozoon ctenopharyngodoni
Cichlidogyrus halli
Cichlidogyrus sclerosus
Paradiplozoon opsariichthydis
Enterogyrus malmbergi
Euryhaliotrema johnii
Ancyrocephalus mogurndae
Thaparocleidus asoti
Thaparocleidus varicus
Microcotylidae
Diplozoidae
Microcotylidae
Dactylogyridae
Gyrodactylidae
Capsalidae
Tetraonchoididae
Tetraonchidae
Diplectanidae
Diplozoidae
Dactylogyridae
Diplozoidae
Dactylogyridae
Mazocraeidea
Dactylogyridea
Gyrodactylidea
Capsalidea
Tetraonchidea
Dactylogyridea
Mazocraeidea
Dactylogyridea
Mazocraeidea
Dactylogyridea
28S_BI
Tree scale: 1

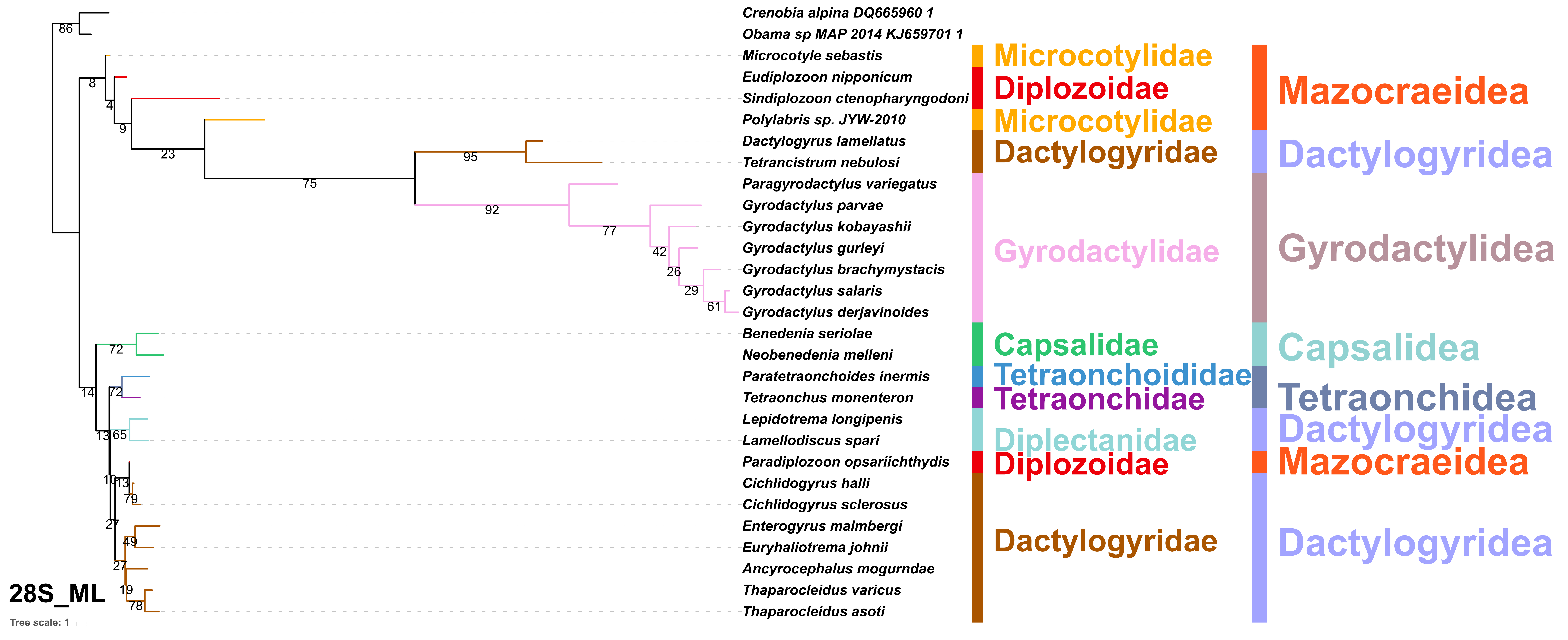

Crenobia alpina DQ665960 1
Obama sp MAP 2014 KJ659701 1
Microcotyle sebastis
Eudiplozoon nipponicum
Sindiplozoon ctenopharyngodoni
Polylabris sp. JYW-2010
Dactylogyrus lamellatus
Tetrancistrum nebulosi
Paragyrodactylus variegatus
Gyrodactylus parvae
Gyrodactylus kobayashii
Gyrodactylus gurleyi
Gyrodactylus brachymystacis
Gyrodactylus salaris
Gyrodactylus derjavinoides
Benedenia seriolae
Neobenedenia melleni
Paratetraonchoides inermis
Tetraonchus monenteron
Lepidotrema longipenis
Lamellodiscus spari
Paradiplozoon opsariichthydis
Cichlidogyrus halli
Cichlidogyrus sclerosus
Enterogyrus malmbergi
Euryhaliotrema johnii
Ancyrocephalus mogurndae
Thaparocleidus varicus
Thaparocleidus asoti
Microcotylidae
Diplozoidae
Microcotylidae
Dactylogyridae
Gyrodactylidae
Capsalidae
Tetraonchoididae
Tetraonchidae
Diplectanidae
Diplozoidae
Dactylogyridae
Mazocraeidea
Dactylogyridea
Gyrodactylidea
Capsalidea
Tetraonchidea
Dactylogyridea
Mazocraeidea
Dactylogyridea
28S_ML
Tree scale: 1
