# Supplementary material for: Mitochondrial Genomes of Two Thaparocleidus Species (Platyhelminthes: Monogenea) Reveal the First rRNA Gene Rearrangement among the Neodermata
Source: Int J Mol Sci. 2019 Aug 28;20(17):4214. doi: 10.3390/ijms20174214 (PMC6747449; doi:10.3390/ijms20174214)
Supplement: Supplementary file 1 [file ijms-20-04214-s001.zip › Supplementary Table S4.docx]

| **Fragment** | **Gene or** | **Primer** | **Sequence (5’-3’)** | **Length** |
| --- | --- | --- | --- | --- |
| **No.** | **region** | **name** |  | **(bp)** |
| ***Thaparocleidus varicus*** | | | |  |
| F1 | *16S* | QYF1 | KTTACCTTTTGCATCATGAT | *835* |
|  |  | QYR1 | HCCGGTCTTAACTCAACTCAT |  |
| F2 | *16S-COX3* | QYF2 | GTTACCTTAGGGATAACAGGG | 1763 |
|  |  | QYR2 | CAAGATGTAATCAAAGAAAC |  |
| F3 | *COX3* | QYF3 | GWCTTGATTACCTATTTATAAAGC | 608 |
|  |  |  |  |  |
|  |  | QYR3 | CNACWAAATGCCANTATCA |  |
| F4 | *COX3-ND4* | QYF4 | CATTTTACTCATGTTGTTATAGG | 2237 |
|  |  | QYR4 | TCTAATTCTTGGCCAGATGC |  |
| F5 | *ND4* | QYF5 | CCWTTACCYCCHTTTCAYTC | 482 |
|  |  | QYR5 | GTAGGAGGAAAWGAAGMTAC |  |
| F6 | *ND4-ATP6* | QYF6 | CCATAGTTGGAAGTCGAAATTG | 914 |
|  |  | QYR6 | CTTCTTGTCCCCACCACAGC |  |
| F7 | *ATP6* | QYF7 | WTDCCTYCTGGTACTCCTTT | 230 |
|  |  | QYR7 | AACCAATGAACTATAGCAAC |  |
| F8 | *ATP6-ND1* | QYF8 | GCTGTGGTGGGGACAAGAAG | 1454 |
|  |  | QYR8 | AATGAACAATAGAATAAGG |  |
| F9 | *ND1* | QYF9 | CGAAAGGGDCCHAATAAGGTWGG | 709 |
|  |  |  |  |  |
|  |  | QYR9 | CRTAACGAACACGAGGTAATG |  |
| F10 | *ND1-COX1* | QYF10 | GGCTTGATTTAGTTCAGTGG | 1276 |
|  |  | QYR10 | GAAACACCAGCTAAATGCAGG |  |
| F11 | *COX1* | QYF11 | GGDTGGACTTTTTATCCTCC | 953 |
|  |  | QYR11 | CAAACACGTCGNGGTAAACCAC |  |
| F12 | *COX1-COX2* | QYF12 | GGTGATGGCCGATGATTAGGG | 865 |
|  |  | QYR12 | GAATAACATCAGAAGAAGTTAC |  |
| F13 | *COX2-ND5* | QYF13 | GAGTATGACTCTTATATGAC | 1580 |
|  |  | QYR13 | CATAGCTTCCAATAATCAAC |  |
| F14 | *ND5* | QYF14 | STTCHCGTTTTGGTGATGTAGG | 211 |
|  |  | QYR14 | CCHGCAGYWACTAAAGTWGAAG |  |
| F15 | *ND5-16S* | QYF15 | GAAGTTGATTATTGGAAGC | 2032 |
|  |  | QYR15 | CAAGTTATCTTCATTAACG |  |
| ***Thaparocleidus asoti*** | | | |  |
| F1 | *COX3* | XEF1 | GTCTTGACTTCCTATATATAAAGC | 608 |
|  |  | QYR1 | CNACWAAATGCCANTATCA |  |
| F2 | *COX3-CYTB* | XEF2 | GTTGGTCTTCATTTTAGTCATG | 647 |
|  |  | XER2 | GATGTTAAAACTGTTGCTGCTC |  |
| F3 | *CYTB* | XEF3 | GTTATAGAAAGGTTATGGTTTG | 498 |
|  |  | XER3 | CAGGCTTAATAGAARCAGGWG |  |
| F4 | *CYTB-ND4* | XEF4 | GTTTTCTTTCATAGTTACTATAG | 1528 |
|  |  | XER4 | TCTAATTCTTGCCCAGAAGC |  |
| F5 | *ND4* | XEF5 | CCTTTRCCYCCHTTTCATTC | 482 |
|  |  | XER5 | GTTGGDGGAAAAGAAGMWAC |  |
| F6 | *ND4-ND1* | XEF6 | CTATGGTTGGTAGACGTAATTG | 2435 |
|  |  | XER6 | CATCAACAACCTATAGCCAG |  |
| F7 | *ND1* | XEF7 | CGTAAGGGACCTAAWAAGGTWGG | 709 |
|  |  | XER7 | CRTAACGHACACGHGGTAATG |  |
| F8 | *ND1-COX1* | XEF8 | CACTTGTGTATTTGCTTGTG | 1348 |
|  |  | XER8 | AACTACTGACTGGATCGTGC |  |
| F9 | *COX1* | XEF9 | GGTTGAACATTTTATCCWCC | 953 |
|  |  | XER9 | CATACACGTCGAGGTAAACCAC |  |
| F10 | *COX1-COX2* | XEF10 | GGGTTCTTATACTAGTGTAG | 894 |
|  |  | XER10 | GAATGAATAACATCAGAAGAAG |  |
| F11 | *COX2* | XEF11 | TTGGACGTCAATGATATTGR | 214 |
|  |  | XER11 | ATVCGGCCDGGAATAGCATC |  |
| F12 | *COX2-ND5* | XEF12 | GTTGATAATCCTTTAGTG | 1581 |
|  |  | XER12 | GTAGAAGAATGAACTAAAC |  |
| F13 | *ND5* | XEF13 | GCTACTTTAATAACTTCTCG | 224 |
|  |  | XER13 | CCHGCAGYAACTAAAGTAGAAG |  |
| F14 | *ND5-16S* | XEF14 | GTTGCTTTAATTGTAGTGAC | 5042 |
|  |  | XER14 | CAAGTTATCTACATTAACGAC |  |
| F15 | *16S* | XEF15 | TTTACCTTTTGCATCATGAT | 835 |
|  |  | XER15 | CCGGTCTTAACTCAACTCAT |  |
| F16 | *16S-COX3* | XEF16 | GTTGCTACCTCGATGTTGAC | 1988 |
|  |  | XER16 | ACCTATCTAAGTAACGCAC |  |
